# Physical health and fitness index in Chinese adolescents: a four-year cross-sectional study in Shandong Province

**DOI:** 10.3389/fpubh.2026.1694201

**Published:** 2026-03-02

**Authors:** Linlin Huang, Junxiang Huang, Shiling Wei, Rui Guo

**Affiliations:** School of Youjiang Medical College for Nationalities of Public Health, Baise, Guangxi, China

**Keywords:** adolescents, physical fitness changes, body mass index, generalized additive model, Shangdong

## Abstract

**Introduction:**

To explore the relationship between physical health and physical fitness index (PFI) in adolescents aged 10 to 19 in Shandong Province. We aimed to offer evidence to support the local implementation of the *National Student Physical Health Standard*.

**Methods:**

Data were obtained from 51,764 adolescents aged 10 to 19 through the Shandong Student Physical Fitness and Health Surveys conducted in 2015, 2016, 2017, and 2020. Assessment of physical fitness included five core items: 50 running, sit and reach, standing long jump, 1,000 running/800 running, and pull-ups/sit-ups, which were then used to calculate the overall PFI. Body Mass Index (BMI) is defined according to the thresholds specified in t*he National Student Physical Health Standards (2014 Revision)*, categorized by gender and grade level: underweight, normal weight, overweight/obese. Socioeconomic data come from provincial statistical yearbooks. We used generalized additive models and logistic regression analyses to examine the associations between PFI and BMI and between PFI and the prevalence.

**Results:**

Normal-weight individuals had the greatest fitness, and fitness declined in underweight and overweight groups. There was an inverted “U”-shaped curve relationship between BMI-Z and PFI. With comparable BMI, PFI changes was higher in females than males. The PFI and the prevalence of overweight/obese showed a consistent upward trend over the years.

**Conclusion:**

Females outperformed males in physical fitness, and fitness levels have improved over time. However, the prevalence of overweight/obese continues to rise, indicating a persistent imbalance between muscle and fat. This suggests that the region may be facing a “development–health paradox.”

## 1 Introduction

Adolescent physical health is a key indicator of a nation’s overall public health (1). Among relevant measures, body mass index (BMI) serves as a universally recognized measure for tracking nutritional status and plays an important role in ensuring health at all stages of life (2). Adolescence, defined by the World Health Organization (WHO) as ages 10–19 years, is a period of significant developmental change (3). In recent years, with China’s socioeconomic transition and changing lifestyles, adolescents have faced increasing challenges such as declining physical fitness, reduced physical activity, and a rising prevalence of overweight/obese (4).

Many countries have investigated the relationship between weight status and physical fitness. Studies in Western populations have shown that physical fitness declines as obesity increases, a pattern that is also observed among children and adolescents in China (5). Research has further demonstrated that adolescent physical fitness is associated with cardiometabolic disease risk, obesity, mental health, cognition, cardiorespiratory fitness (CRF), and musculoskeletal fitness (MSF) (1). Due to substantial differences in economic development, living conditions, and educational practices across provinces, understanding regional patterns in China is essential. As one of China’s most populous provinces, Shandong saw the adolescent overweight and obesity rate increase from 12.3% in 2010 to 18.7% in 2020 (6), a trend consistent with the national average annual rise in adolescent BMI. Studies have confirmed that insufficient physical fitness training contributes to this alarming increase in overweight/obese among adolescents (7).

Previous studies have identified adolescence as a critical period for physical health development, characterized by strong continuity and predictive value for adult health outcomes (1, 31). The physical fitness indicator (PFI), a composite index that reflects cardiorespiratory function, muscular strength, and flexibility, has gained increasing attention in sports medicine due to its strong association with physical health and its utility in assessing adolescent fitness levels (8–10). Research has shown that BMI and physical fitness exhibit a negative quadratic relationship (11). Kwieciński found that BMI and PFI exhibit an inverted U-shaped relationship in adults, where moderate BMI confers the best physical performance, and both extremes are unfavorable. Whether this pattern holds for adolescents is uncertain, as rapid changes in height, muscle mass, fat distribution, and hormonal status during adolescence may alter the shape or position of the BMI–fitness curve (12).

For these reasons, we aimed to assess the secular trends in physical health among adolescents aged 10–19 years in Shandong Province from 2015 to 2020, examine the association between BMI and PFI, and explore the region potential strategies to optimize the implementation of the *National Student Physical Health Standard* (13).

## 2 Materials and methods

### 2.1 Participants

Data come from the Adolescent Health Thematic Database in the Population Health Data Archive (PHDA) (14). We used physical fitness data from 51,764 adolescents aged 10 to 19 years, collected from 186 middle schools in 17 cities across Shandong Province, China. After excluding individuals with missing data or biologically implausible values, valid data were retained from surveys conducted in 2015 (*n* = 11,291), 2016 (*n* = 2,408), 2017 (*n* = 17,140), and 2020 (*n* = 20,925). Although the study targeted adolescents aged 10–19 years, a very small number of participants fell slightly outside this age range due to grade-based sampling. These cases were retained because they belonged to the same school grade and met the inclusion criteria. Body Mass Index (BMI = weight (kg) / [height (m)] ^2^) is defined according to the thresholds specified in the *National Student Physical Health Standards* (*2014 Revision*) (13) and categorized by gender and grade level as underweight, normal weight, and overweight/obese.

### 2.2 Physical fitness

We measured five core components: 50 m running, sit and reach, standing long jump, 1,000 m running for males or 800 m running for females, and pull-ups for males or sit-ups for females, each representing a distinct aspect of physical fitness. These items are core components of the National Student Physical Health Standard and reflect major dimensions of physical fitness, including speed, flexibility, explosive power, muscular strength, and cardiorespiratory endurance. The PFI based on these five tests has been previously validated in Chinese adolescents, and studies have demonstrated its reliability and its ability to represent overall fitness^[7–9]^. Five core items were standardized into Z-scores, and the PFI was calculated as the sum of five Z-scores: PFI = Z standing long jump + Z sit and reach + Z pull-ups (male)/1 min sit-ups (female) - Z 50 m running - Z 1000 m running (male)/800 m running (female) (8–10). Z-scores were calculated using pooled means and standard deviations across 2015–2017 and 2020, separately by sex. For the 50 m, 1,000 m, and 800 m runs, Z-scores were reversed because shorter times indicate better fitness. A higher PFI score indicates better physical fitness. A higher PFI score indicates better physical fitness. PFI was divided into three categories based on the 25th (P25), 50th (P50), and 75th (P75). For males, the thresholds were P75 = −1.2765, P50 = −0.1607, and P25 = 1.0535; for females, P75 = −1.0821, P50 = 0.0412, and P25 = 1.1568. Low fitness was defined as PFI ≤ P25, moderate fitness as PFI between P25 and P75, and high fitness as PFI > P75.

### 2.3 Socioeconomic data

Socioeconomic data come from the *Shandong Statistical Yearbook* (2015–2017, 2020) (15). We applied the World Bank’s per capita gross domestic product (GDP) classification method to group cities into three development tiers: High-tier, cities with per capita GDP consistently above the national average; Mid-tier, cities with GDP levels fluctuating around the national average; and Low-tiers, cities with per capita GDP consistently below the national average.

### 2.4 Statistical analysis

We used descriptive statistics to examine physical fitness trends across different BMI categories, using standardized PFI values. Differences in physical fitness composition across categorical groups were examined using χ^2^ test. Group differences across BMI categories were analyzed using one-way ANOVA with Dunnett’s *post hoc* test, or the Kruskal–Wallis rank-sum test with Bonferroni correction for multiple comparisons. We used the mgcv package to perform generalized additive model analysis with BMI Z-scores treated as a smooth and independent variable to examine their relationship with PFI. To further evaluate whether the sampling change could bias the trend analysis, we added an interaction term between year and BMI_Z. We used MASS package to do logistic regression analyses to assess the prevalence trends of underweight, normal weight, and overweight/obese in relation to the PFI. The models were adjusted for age, sex, and inner-province socioeconomic status. All statistical analyses were conducted using R (version 4.4.2).

## 3 Results

A total of 51,764 adolescents were included in the analysis, with a mean age of 14.29 ± 2.50 years, including 33,896 females (65.5%) and 17,868 males (34.5%). Participants included 2,776 underweight (5.36%), 37,854 normal weight (73.13%), and 11,134 overweight/obese (21.51%). Significant differences in PFI were observed across age, weight status categories, and regional per capita GDP (*p* < 0.01) (Table 1).

**Table 1:** General physical fitness of adolescents in Shandong Province.

Groups	Low-fitness (*N* = 12,940)	Moderate –fitness (*N* = 25,884)	High-fitness (*N* = 12,940)	*χ^2^*	*p*
Sex				0.000	1.000
Males (*N* = 17,868)	4,467 (25.0)	8,935 (50.0)	4,466 (25.0)		
Females (*N* = 33,896)	8,473 (25.0)	16,949 (50.0)	8,474 (25.0)		
Age group^a^ (years)				837.460	<0.001
8- (*N* = 4)	1 (25.0)	0 (0.0)	3 (75.0)		
10- (*N* = 28,095)	8,426 (30.0)	13,295 (47.3)	6,374 (22.7)		
15- (*N* = 23,618)	4,505 (19.1)	12,568 (53.2)	6,545 (27.7)		
20- (*N* = 47)	9 (19.1)	24 (51.1)	14 (29.8)		
Physical condition				68.123	<0.001
Underweight (*N* = 2,776)	873 (31.4)	1,285 (46.3)	618 (22.3)		
Normal (*N* = 37,854)	9,301 (24.6)	18,969 (50.1)	9,584 (25.3)		
Overweight/Obese (*N* = 11,134)	2,766 (24.8)	5,630 (50.6)	2,738 (24.6)		
GDP per capita (¥)				557.937	<0.001
High-tier (*N* = 16,647)	5,081 (30.5)	8,255 (49.6)	3,308 (19.9)		
Mid-tier (*N* = 25,003)	5,621 (22.5)	12,546 (50.2)	6,836 (27.3)		
Low-tier (*N* = 10,114)	2,239 (22.1)	5,082 (50.2)	2,792 (27.6)		

a Age groups include a small number of students slightly outside the 10–19 age range due to grade-based sampling structure.

**Figure 1:**
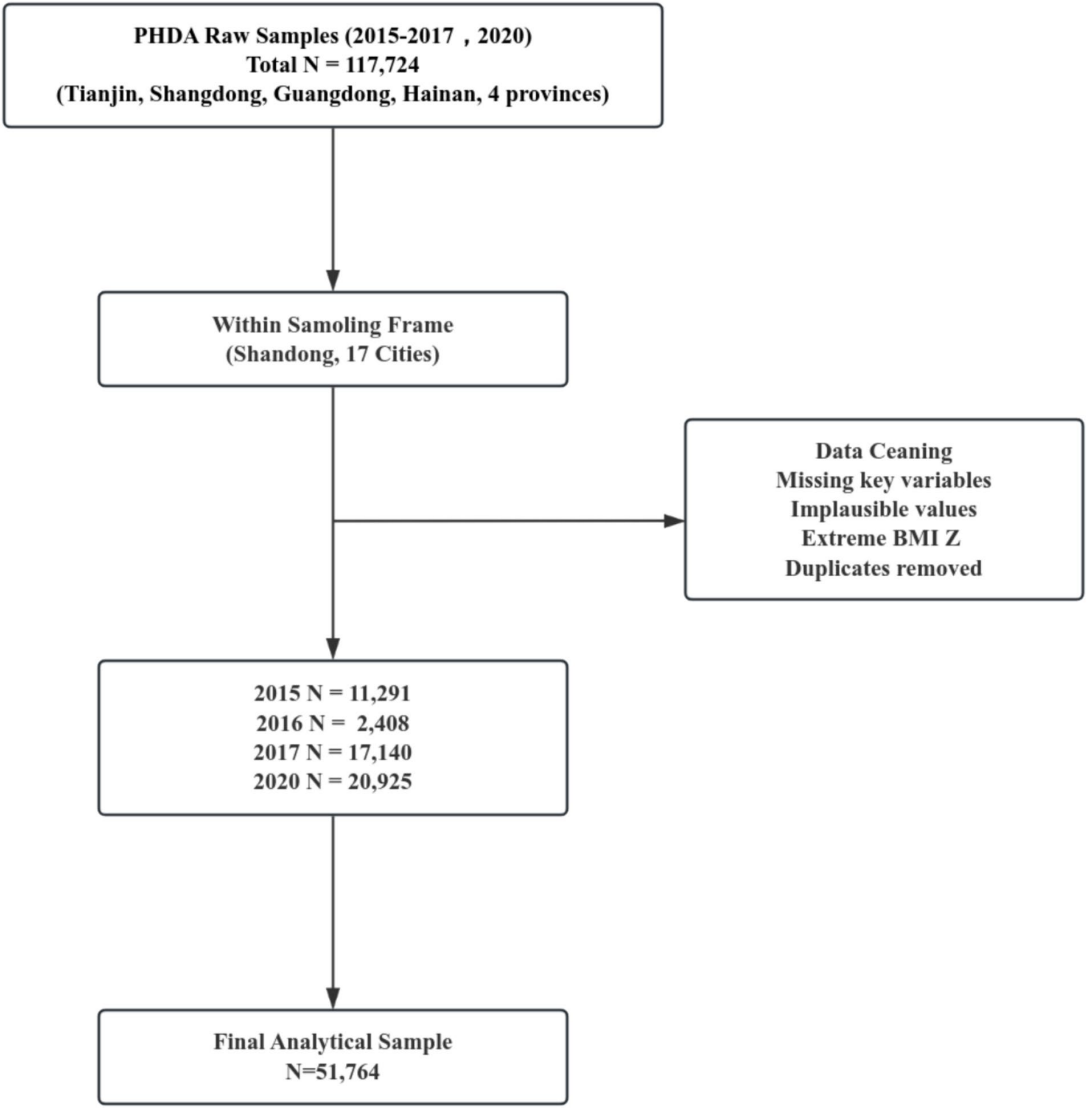
Flowchart of PHDA data screening and final sample construction (2015–2017, 2020).

PFI differed significantly across BMI categories by sex (*p* < 0.01). The mean PFI for normal weight males was −0.032, higher than that for underweight (−0.272) and overweight/obese (−0.139); all pairwise comparisons were statistically significant (*p* < 0.01). The mean PFI for normal weight females was −0.047, higher than that for underweight (−0.307) and overweight/obese (−0.098). The comparison between underweight and normal-weight females revealed a significant difference (*p* < 0.01). PFI differed significantly across BMI categories in both the 10–14 and 15–19 age groups (*p* < 0.01), with normal weight individuals showing higher PFI than those underweight or overweight/obese. All pairwise comparisons were statistically significant (*p* < 0.01) (Figure 1).

In high-tier regions, the mean PFI for normal weight individuals (−0.244) was lower than in mid-tier (0.212) and low-tier regions (0.185). Significant PFI differences across BMI categories were found in both high- and mid-tier regions (*p* < 0.01), specifically between underweight and normal weight groups. In low-tier regions, the difference between normal weight and overweight/obese groups was also significant (*p* < 0.01) (Table 2).

**Table 2:** Physical fitness by BMI category among adolescents in Shandong Province.

PFI	BMI			Statistic	*p*
	Underweight (*N* = 2,776)	Normal (*N* = 37,854)	Overweight/obese (*N* = 11,134)		
Sex					
Males (*N* = 17,868)	−0.272 ± 1.885^b^	−0.032 ± 1.837	−0.139 ± 1.878^b^	31.068^c^	<0.001
Females (*N* = 33,896)	−0.307 ± 2.013^b^	−0.047 ± 1.836	−0.098 ± 1.880	57.182^c^	<0.001
Age group (years)					
8- (*N* = 4)	2.696 ± 0.000	−0.791 ± 2.959	—	2.667^c^	0.102
10- (*N* = 28,095)	−0.506 ± 2.126^b^	−0.182 ± 1.965	−0.111 ± 1.983^b^	47.000^c^	<0.001
15- (*N* = 23,618)	0.011 ± 1.652^b^	0.247 ± 1.654	0.160 ± 1.718^b^	33.223^c^	<0.001
20- (*N* = 47)	0.721 ± 1.552	0.132 ± 1.441	0.327 ± 1.657	0.409^d^	0.667
GDP per capita (¥)					
High-tier (*N* = 16,647)	−0.665 ± 1.893^b^	−0.244 ± 1.775	−0.276 ± 1.808	49.775^c^	<0.001
Mid-tier (*N* = 25,003)	−0.220 ± 1.988^b^	0.212 ± 1.945	0.122 ± 1.854	46.725^c^	<0.001
Low-tier (*N* = 10,114)	0.021 ± 1.934	0.185 ± 1.843	0.044 ± 1.797^b^	9.590^c^	0.008

b Underweight, normal weight, overweight/obese pairwise comparisons, *p* < 0.01.

c *F*-value.

d *H*-value.

**Figure 2:**
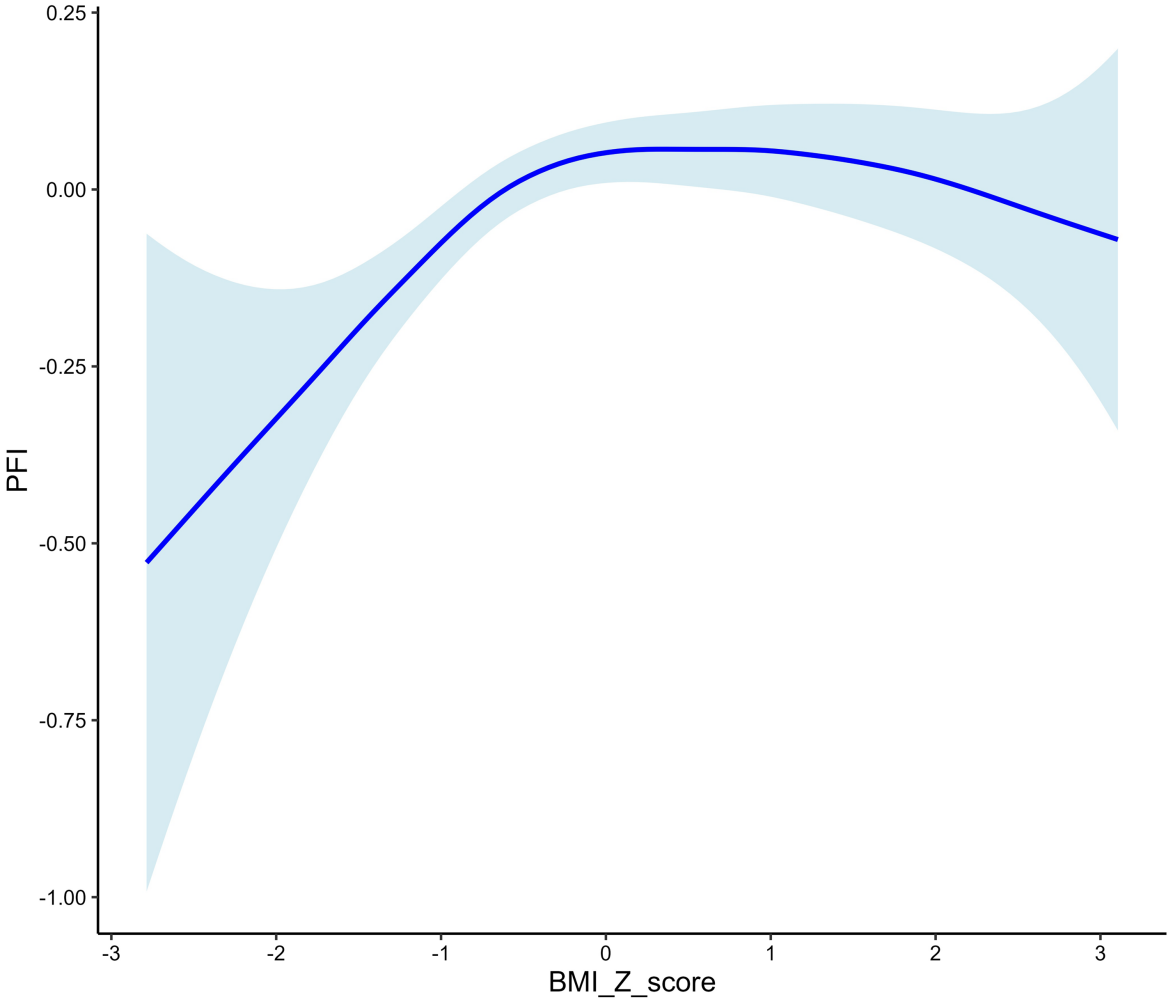
Relationship between PFI and BMI Z score.

**Figure 3:**
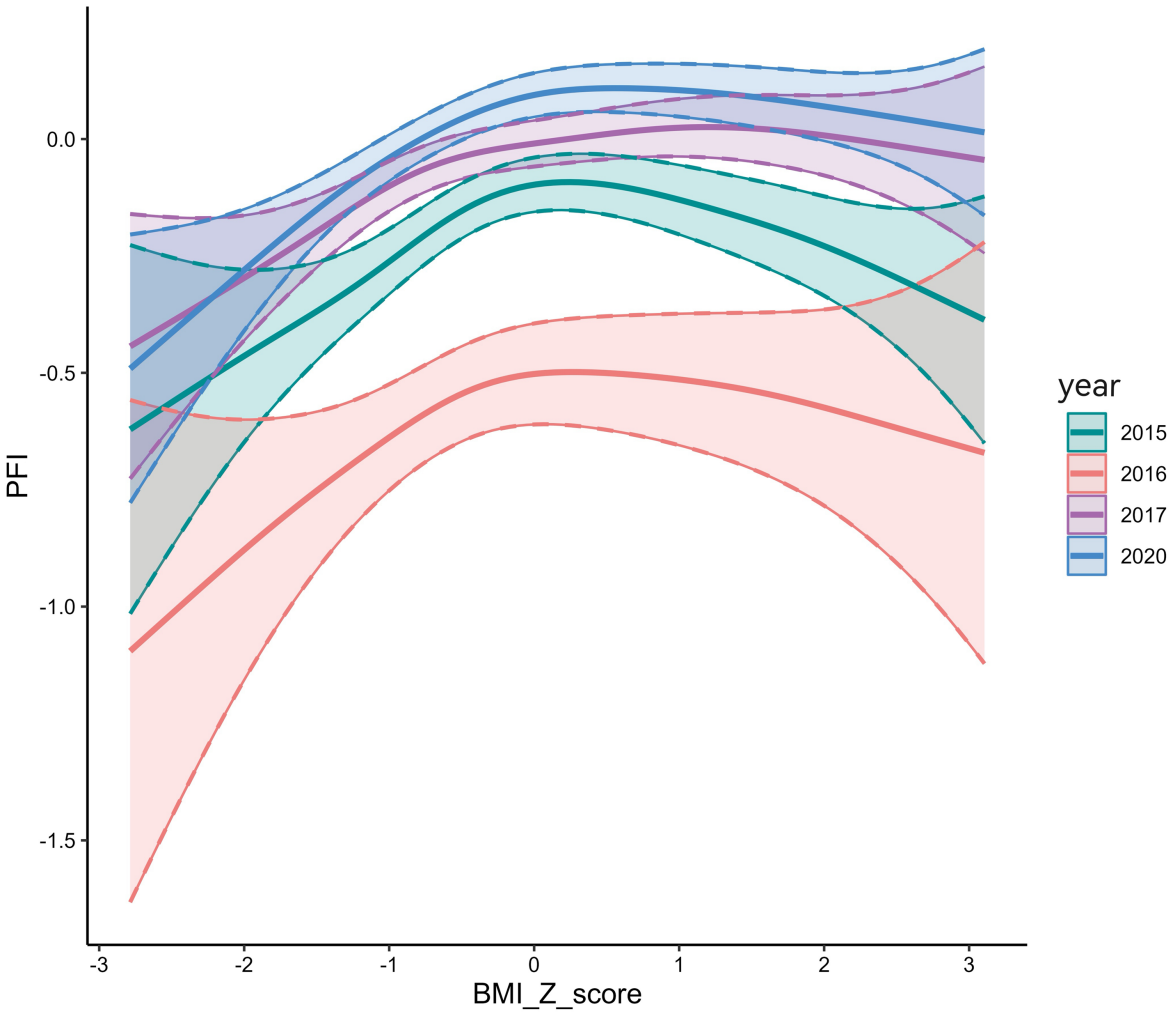
Relationship between PFI and BMI Z scores (2015–2020).

**Figure 4:**
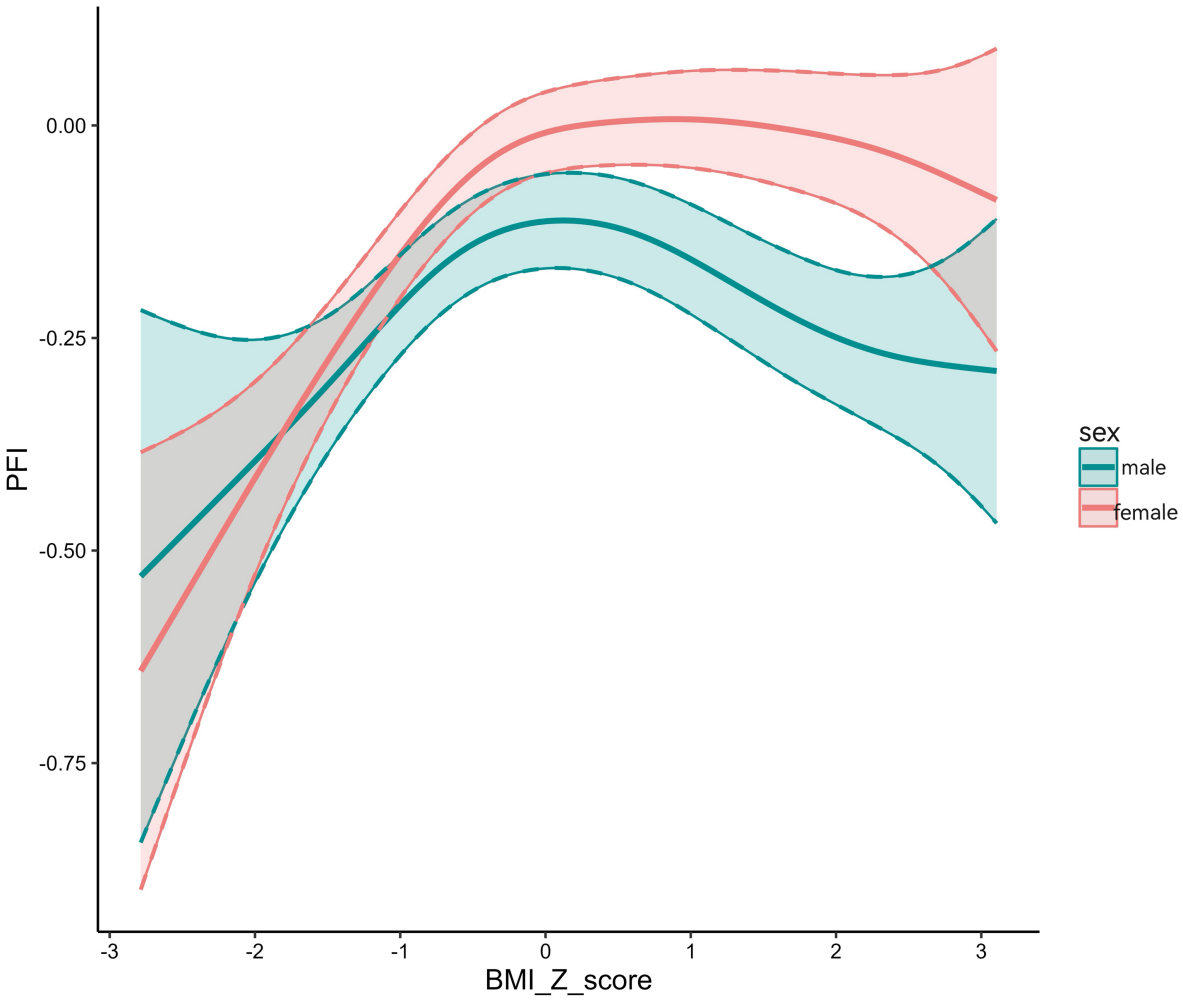
Relationship between PFI and BMI *Z* scores by gender.

PFI exhibited an inverted U-shaped association with BMI Z-scores, reaching its maximum at a Z-score of 0 (Figure 2). Over time, the PFI–BMI Z-score curve shifted upward and rightward. In 2020, the curve was highest, with the peak furthest to the right, indicating higher PFI at the same BMI Z-score than in previous years (Figure 3). BMI Z-scores had a stronger effect on PFI in females, with a steeper U-shaped curve compared to the flatter curve in males. For example, the peak PFI is −0.06 for males at a BMI Z-score of 0.8, and 0.04 for females at −0.5 (Figure 4).

**Figure 5:**
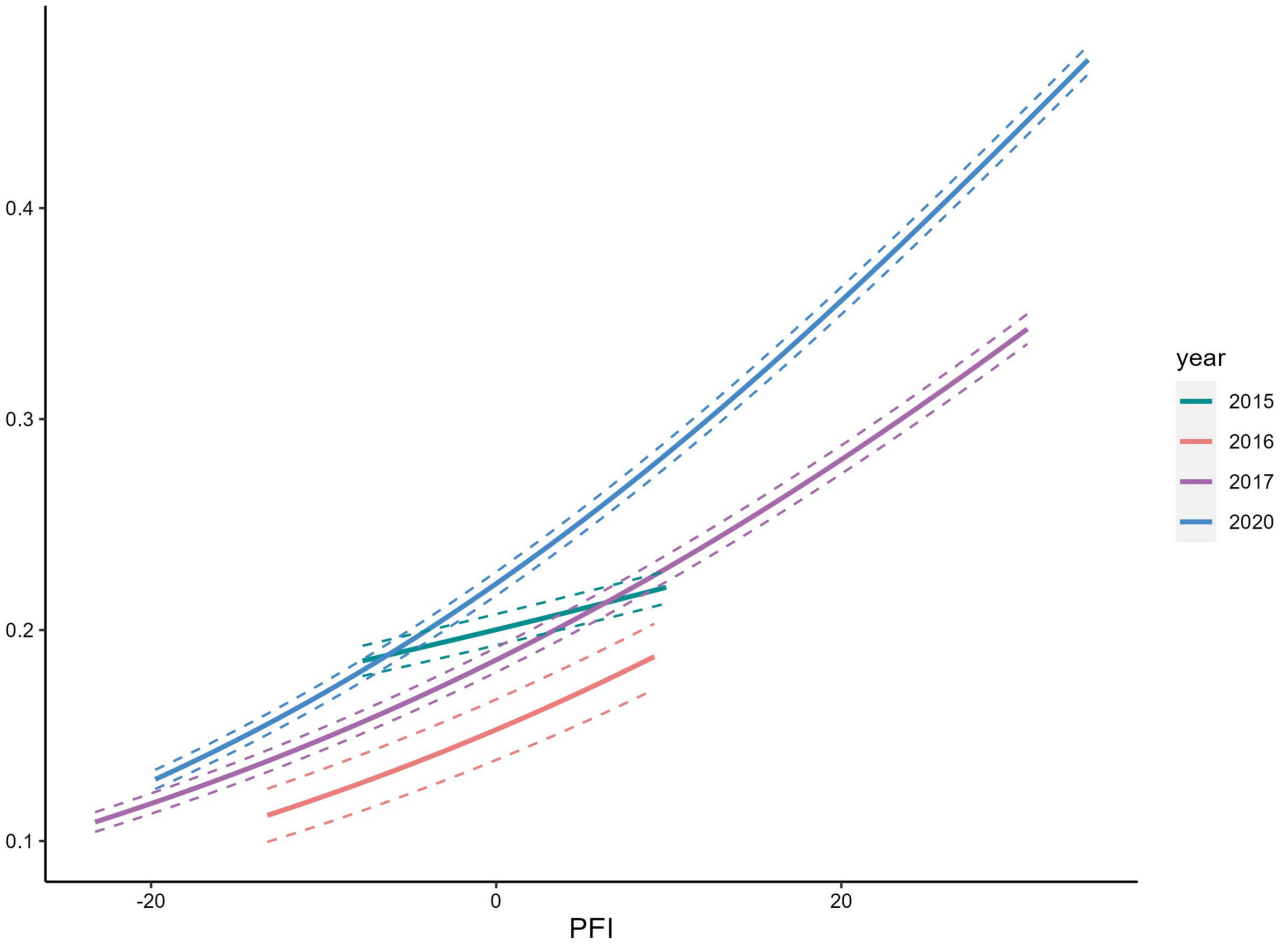
Relationship between overweight/obese rate and PFI.

**Figure 6:**
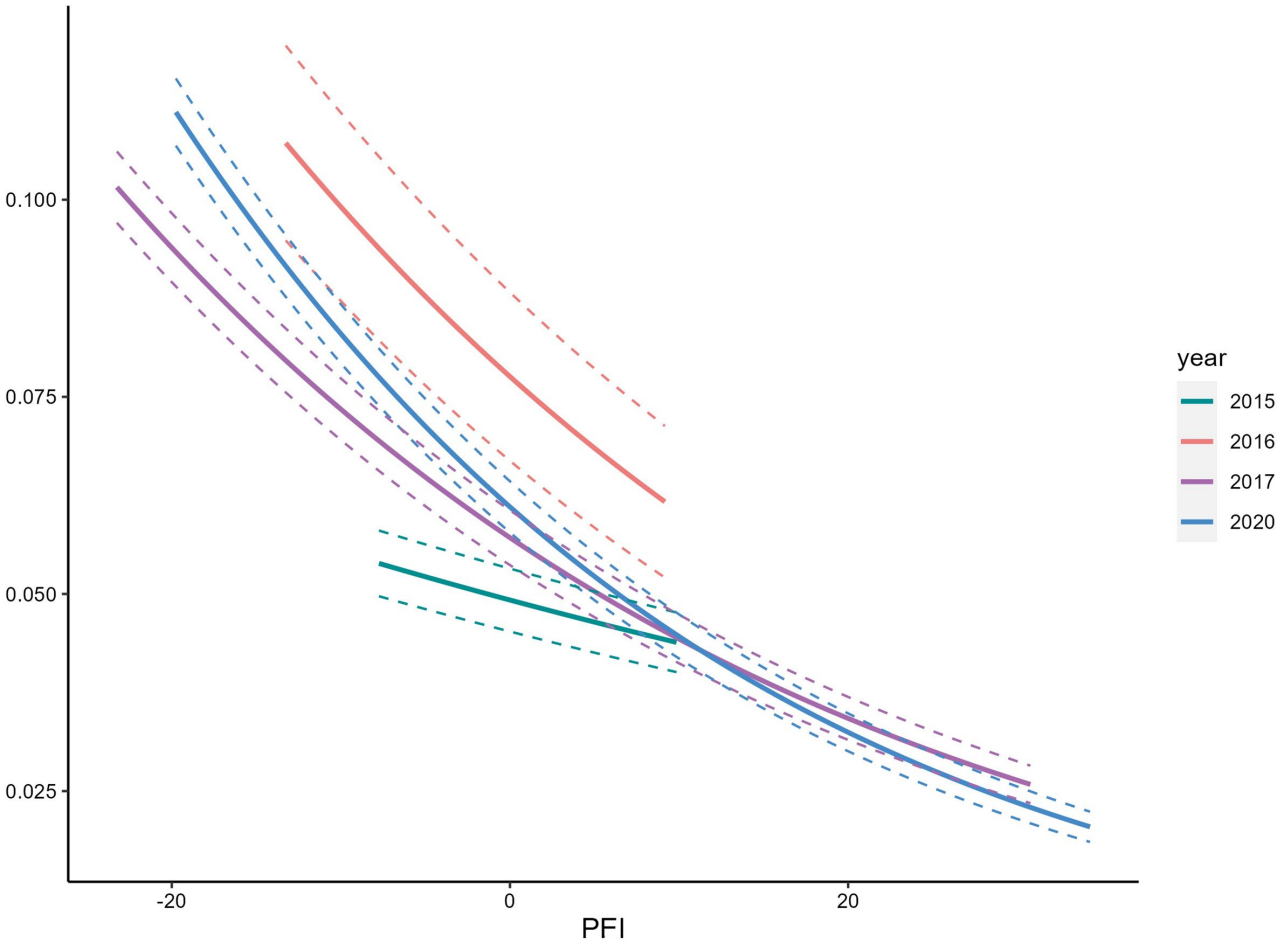
Relationship between low body weight rate and PFI.

**Figure 7:**
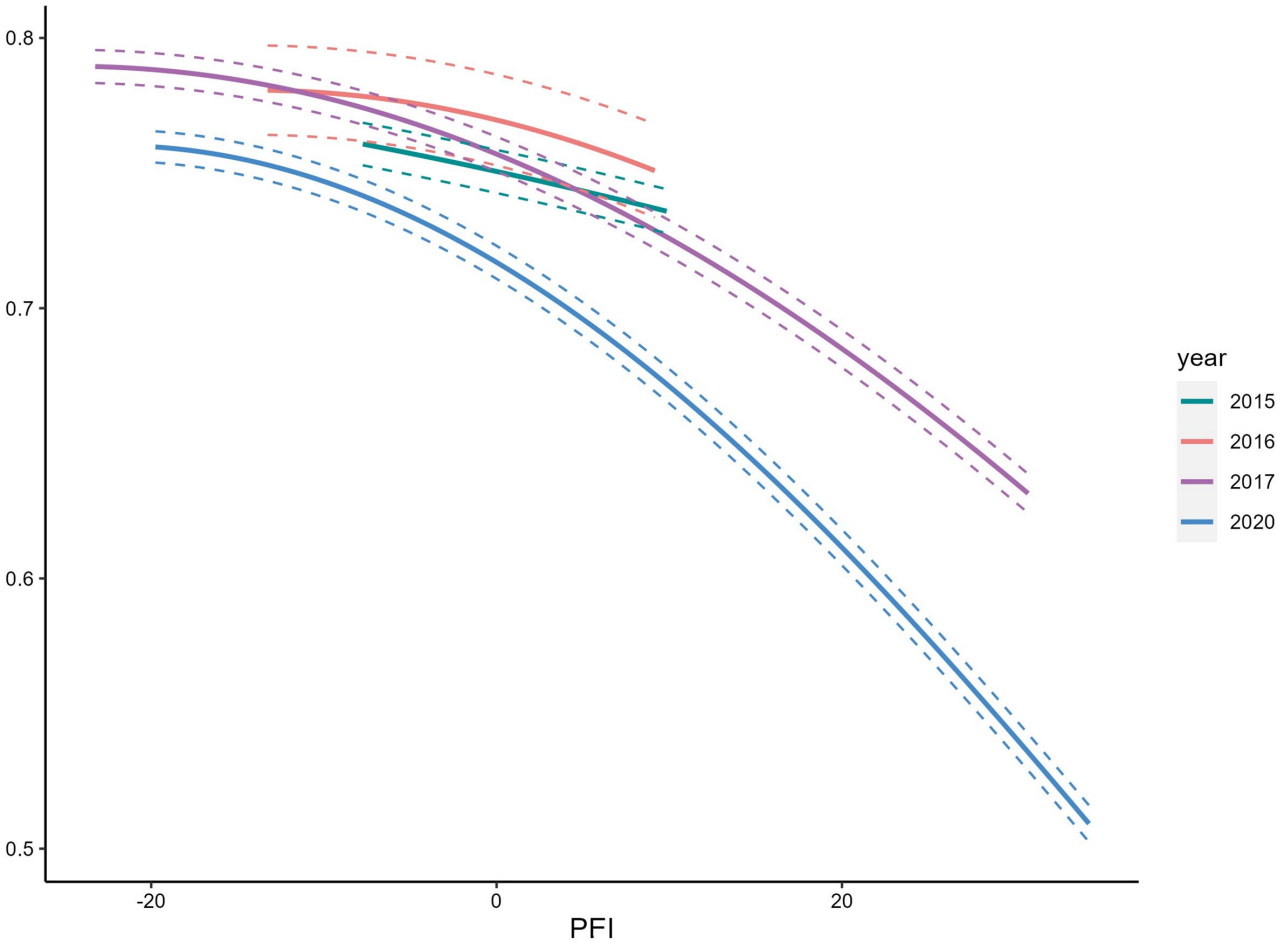
Relationship between normal body mass rate and PFI.

We observed a positive association between PFI and overweight/obese (Figure 5), and a negative association with underweight (Figures 6, 7). Among s adolescents with lower physical fitness (PFI < 0), the association between PFI and underweight changed substantially from 1985 to 2014, while the association with overweight/obese remained relatively stable. That is, adolescents with low PFI were more likely to be underweight than overweight/obese in 2015. Over time, the prevalence of underweight declined, while overweight and obesity became increasingly common.

## 4 Discussion

Our analysis of data from 51,764 adolescents in Shandong Province from 2015 to 2020 showed that the mean PFI among normal-weight adolescents was lower than that reported in Japan (16), as well as in Nanjing (17), Henan (18), Guangdong (19), and Guangxi Zhuang Autonomous Region in China (9). Moreover, adolescents with normal weight showed higher PFI than those who were underweight or overweight/obese. This finding is consistent with a previous correlation study on physical fitness conducted in Shandong Province (20). A retrospective analysis of 1.5 million Chinese students (21) also reached a similar conclusion, indicating that both low and high BMI may have negative effects on physical fitness in adolescents.

In males, PFI differed significantly from normal weight in both underweight and overweight/obese groups. In females, only the underweight group differed significantly from normal weight, with no difference observed in the overweight/obese group. These findings suggest sex differences in physical fitness across BMI categories (8). Physical fitness showed little variation between the normal weight and overweight/obese females. This may be because overweight/obese females are more influenced by sociocultural body ideals than males, leading to greater concern about body shape and appearance (22). As a result, they may be more likely to engage in physical activity to improve their physique and maintain a healthy weight, thereby enhancing their fitness performance.

Our study found an inverted U-shaped association between adolescent PFI and BMI Z-scores. Females exhibited better PFI performance than males at comparable BMI Z-scores. This indicates that females may maintain physical fitness more effectively across BMI levels. Several mechanisms support this observation. One possible explanation lies in physiological differences. Estrogen promotes the accumulation of subcutaneous fat in the hips and thighs in females, which can serve as an energy reserve during endurance exercise. In contrast, androgens increase visceral fat deposition in males, which may contribute to heightened inflammatory responses and negatively impact physical performance (21). Indicator selection may also introduce bias. For example, the sit-and-reach test, a measure of flexibility, may overestimate overall physical fitness in females (23). A third factor is the influence of cultural norms. Sociocultural pressure around body image may lead to body shape anxiety in females, prompting earlier development of health management awareness. This, in turn, encourages regular physical activity aimed at improving appearance and may indirectly enhance physical fitness performance (22). This interpretation aligns with findings reported by Yuqiang Li et al. (16). Furthermore, large-scale epidemiological studies have consistently shown sex differences in fitness indicators throughout adolescence, reinforcing the plausibility of our results (24).

Previous studies have shown that adolescents in high-GDP regions tend to have lower physical fitness than those in low-GDP regions (4), a pattern also observed in our study. Additionally, the study found that in high-GDP regions, differences in body size between overweight/obese and normal-weight adolescents had little impact on physical fitness, suggesting that the influence of economic development may outweigh individual differences in body composition. Such differences in physical fitness may be related to factors such as social behavior patterns associated with economic level (25) and differences in dietary structure (26). For example, n high-GDP regions, physical activity tends to decrease, commuting is more dependent on private vehicles, and the intake of processed and energy-dense foods has increased (27, 28). In contrast, low-GDP regions are characterized by labor-intensive lifestyles, with students more likely to walk or cycle to school. Traditional dietary patterns in these areas may be associated with nutritional imbalance, such as excessive carbohydrate intake and insufficient protein consumption (27, 28). In summary, economically developed regions may be facing a “development–health paradox.” International studies show that economic growth and better healthcare do not automatically improve physical fitness. WHO and evidence from many countries report that rapid urbanization reduces physical activity, increases sedentary time, and raises intake of processed foods. These changes contribute to higher adolescent obesity even when public health conditions improve. In our study, the economically developed areas of Shandong Province had lower PFI levels. This suggests that modern lifestyles may offset the health gains of economic development. It also highlights the importance of regional health differences. Further research is needed to quantify the dietary patterns and behavioral factors of adolescents in these areas, in order to better explain why economic growth has not been accompanied by improvements in physical fitness.

From 2015 to 2020, overall physical fitness levels improved, while the prevalence of overweight and obesity also increased. The upward shift and rightward movement of the fitness–BMI curves indicate enhanced fitness performance, whereas the body composition trends show a more rapid rise in overweight and obesity. This suggests that adolescents may face worsening adiposity even as their fitness improves. This paradoxical pattern, in which fitness increases but obesity continues to rise, differs from national trends in China (29) and may be partly attributable to recent changes in school physical education policies in Shandong Province. Since 2015, the province has increased the weight of physical education in high-school entrance examinations and implemented the “one hour of daily school exercise” policy, encouraging greater participation in structured physical activity and improving fitness test results. However, dietary habits and lifestyle behaviors have not improved correspondingly. Regional surveillance data show increased consumption of high-carbohydrate foods, sugar-sweetened beverages, and processed products, along with greater screen time and reduced active commuting (4, 30). These changes likely result in higher energy intake and insufficient energy expenditure, which continues to drive the rise in obesity. Improvements in fitness may therefore reflect short-term training adaptations rather than genuine enhancements in body composition. This explanation requires further quantitative investigation.

Our study also has several limitations that should be noted. First, it is a cross-sectional survey, which does not allow for causal inference and can only identify associations between BMI and PFI. Second, changes in the sampling design led to differences in sample size, which may have introduced some bias. Stratified cluster sampling was used from 2015 to 2017, while data collection was affected after 2018 due to disruptions caused by the COVID-19 pandemic. PPS sampling was adopted in 2020. Cluster sampling may be influenced by school-level fitness differences, while PPS sampling gives larger schools higher selection probabilities, potentially inflating the 2020 PFI mean. To address this potential bias, we performed sensitivity analyses with interaction terms. The inverted U-shaped relationship between PFI and BMI_Z remained consistent, suggesting that sampling heterogeneity may affect absolute values but does not change the study’s main conclusion. Third, physical fitness is a composite health indicator. Evidence from six consecutive national surveys suggests that composite PFI is a valid measure of physical capacity in adolescents. However, it has not yet been independently validated (4). Finally, data on covariates such as nutrition, exercise routines, and household environment were not collected, though they may have a considerable impact on changes in adolescent physical health and fitness. Future studies should incorporate these behavioral and environmental factors, ideally drawing on established national surveillance systems such as the China Health and Nutrition Survey (CHNS), to better clarify the pathways linking BMI, physical fitness, and adolescent health.

## 5 Conclusion

The study revealed an inverted U-shaped correlation between BMI and PFI among adolescents in Shandong, where normal-weight individuals had the greatest fitness, and fitness declined in underweight and overweight groups. Females generally outperformed males, likely due to physiological differences, sociocultural pressures, and related health behaviors. Economic development appeared to influence fitness more than physical constitution, as adolescents in high-GDP regions showed lower fitness levels, suggesting a possible “development–health paradox.” Fitness improved rapidly while overweight/obese rates also rose, diverging from national patterns between 2015 and 2020. This suggests that current policies (physical education reforms and mandatory exercise) have not effectively improved body composition. Future research should incorporate nutrition, activity, and environmental factors to guide more targeted interventions.

## Data Availability

The datasets presented in this study can be found in online repositories. The names of the repository/repositories and accession number(s) can be found below: https://doi.org/10.12213/11.A0031.202107.209.V1.0.
